# A Mobile Telehealth Intervention for Adults With Insulin-Requiring Diabetes: Early Results of a Mixed-Methods Randomized Controlled Trial

**DOI:** 10.2196/resprot.4035

**Published:** 2015-02-26

**Authors:** Justine Baron, Shashivadan Hirani, Stanton Newman

**Affiliations:** ^1^Institute of Cardiovascular ScienceUniversity College LondonLondonUnited Kingdom; ^2^School of Health SciencesCity University LondonLondonUnited Kingdom

**Keywords:** mobile telehealth, self-management, mixed-method design, diabetes, glycated hemoglobin (HbA1c), health-related quality of life, intervention fidelity, behavior change

## Abstract

**Background:**

The role of technology in health care delivery has grown rapidly in the last decade. The potential of mobile telehealth (MTH) to support patient self-management is a key area of research. Providing patients with technological tools that allow for the recording and transmission of health parameters to health care professionals (HCPs) may promote behavior changes that result in improved health outcomes. Although for some conditions the evidence of the effectiveness of MTH is clear, to date the findings on the effects of MTH on diabetes management remain inconsistent.

**Objective:**

This study aims to evaluate an MTH intervention among insulin-requiring adults with diabetes to establish whether supplementing standard care with MTH results in improved health outcomes—glycated hemoglobin (HbA1c), blood pressure (BP), health-related quality of life (HRQoL), diabetes self-management behaviors, diabetes health care utilization, and diabetes self-efficacy and illness beliefs. An additional objective was to explore the acceptability of MTH and patients’ perceptions of, and experience, using it.

**Methods:**

A mixed-method design consisting of a 9-month, two-arm, parallel randomized controlled trial (RCT) was used in combination with exit qualitative interviews. Quantitative data was collected at baseline, 3 months, and 9 months. Additional intervention fidelity data, such as participants’ MTH transmissions and contacts with the MTH nurse during the study, were also recorded.

**Results:**

Data collection for both the quantitative and qualitative components of this study has ended and data analysis is ongoing. A total of 86 participants were enrolled into the study. Out of 86 participants, 45 (52%) were randomized to the intervention group and 36 (42%) to the control group. Preliminary data on MTH training sessions and MTH usage by intervention participants are presented in this paper. We expect to publish complete study results in 2015.

**Conclusions:**

The range of data collected in this study will allow for a comprehensive evaluation of processes and outcomes. The early results presented suggest that MTH usage decreases over time and that MTH participants would benefit from attending more than one training session.

**Trial Registration:**

ClinicalTrials.gov NCT00922376; http://clinicaltrials.gov/ct2/show/NCT00922376 (Archived by WebCite at http://www.webcitation.org/6Vu4nhLI6).

## Introduction

### Overview

Diabetes currently affects approximately 366 million people and this number is expected to increase to 552 million by 2030 [1]. Diabetes care in England is estimated to take up between 5 and 10% of all National Health Service (NHS) expenditures [2]. The difficulties of living with diabetes, with its complex regimen and need for behavior change, is challenging, making good self-management difficult to achieve [3].

Telehealth (TH) offers patients the ability to record diabetes-related information electronically and transfer this to their health care professional (HCP), allowing them to be easily connected over time to HCPs. Using a mobile platform for TH, referred to as mobile telehealth (MTH), enables a transition from a health delivery model where monitoring is infrequent and discrete in clinics, to continuous and potentially nonintrusive monitoring taking place across locations [4]. TH is believed to hold the potential to revolutionize care delivery processes by improving the efficiency and quality of the care [5], enhancing patient experience and health-related quality of life (HRQoL), increasing patient confidence in addressing their needs, and supporting self-management [6]. However, questions remain as to whether TH technologies will be acceptable and useful and lead to improved outcomes in all patients [7,8].

Several systematic reviews have examined the impact of TH in people with diabetes and yielded inconsistent or inconclusive findings [9-16]. Concerns about the quality of the studies in this area have been raised and the need for more robust methodologies emphasized [17-19]. To date, the focus has been on glycated hemoglobin (HbA1c) as a primary outcome and patient-reported outcomes have often not been examined [20]. There is also little research on the factors that influence patients’ engagement with the technology, as well as on the process variables through which TH might impact health outcomes [21,22].

This study aimed to address some of these gaps in the literature. The evaluation in this study was informed by the recommendations made by the Medical Research Council (MRC) [23]. The MRC recommends that evaluations consist of several components, including a review of the literature, the use of theory to guide design and evaluation, and consideration for both outcomes and process variables. The importance of assessing intervention fidelity has also been underlined [24], as has the valuable contribution of qualitative methods to reach a better understanding of the factors that could help explain study findings [23].

### Study Aims

The aim of this randomized controlled trial (RCT) is to evaluate the effectiveness of an MTH intervention on adults with insulin-requiring diabetes. The MTH intervention involved data transmission, feedback, and education. The intervention will be examined based on clinical outcomes—HbA1c and blood pressure (BP)—diabetes health care utilization, HRQoL, and self-management behaviors. Secondary aims are to assess the impact of MTH on process variables, including diabetes self-efficacy and illness beliefs, and to test whether these mediate the potential effects of the intervention on the outcomes. Additional aims include the identification of predictors of MTH usage, the assessment of intervention fidelity, and the exploration of patients’ experiences with, and perceptions of, the acceptability of MTH.

### Theoretical Framework

The theoretical foundations guiding the concepts used in the study included Bandura’s social cognitive theory [25], Leventhal’s model of illness beliefs [26], and Davis’s Technology Acceptance Model (TAM) [27]. These theories propose that self-efficacy and illness beliefs influence health behaviors, and that factors such as perceived usefulness and perceived ease of use determine technology usage. The key behavior change techniques involved in the MTH intervention—self-monitoring with feedback and education—have been related to changes in self-efficacy [28] and education has been linked to changes in beliefs [29].

Concepts from the TAM were selected to examine MTH usage as work using this model has shown that acceptability, which is determined by perceived usefulness and perceived ease of use, predicts usage behavior across a range of technologies. The information technology training provided, and the extent to which the technology is perceived to interfere in life, are two further factors shown to be related to technology usage [30,31], therefore, these factors are also included to address the question on predictors of MTH usage.

## Methods

### Ethical Approval and Registration

This study received full ethical approval from the Joint University College London/University College London Hospitals (UCL/UCLH) Committees on the Ethics of Human Research, Committee Alpha (09/H0715/69). The RCT has been registered with ClinicalTrials.gov (NCT00922376).

### Hypotheses

The primary hypothesis is that standard care supplemented with MTH can achieve greater improvements in HbA1c and BP. Secondary hypotheses are that the intervention will result in greater improvements in self-management behaviors, HRQoL, and psychological well-being compared to standard care. In line with the theories, we further hypothesize that improvements in self-efficacy and illness beliefs will determine the change in health outcomes and that acceptability of MTH, self-efficacy to use MTH, and adequacy of MTH training will significantly predict telehealth usage.

### Study Design

This study used a mixed-method design including a two-arm parallel RCT with repeated measurements—baseline, 3 months, and 9 months—and qualitative exit interviews with patients. A sequential design was used in that the RCT was conducted prior to the qualitative study. As such, the qualitative enquiry was embedded in the quantitative study and aimed to extend and help elucidate the quantitative findings.

### Sample Size Calculation

A sample of 248 participants (124 participants per group) was required to detect significant group differences on the primary outcome, HbA1c, for a two-group and repeated measures design. These calculations were based on equal group sizes, an attrition rate of 30%, 80% power to detect differences at the *P*=.05 significance level (two-sided), with correlations between measurements of .70, and an effect size of 0.21 standard deviation units.

### Trial Population and Recruitment

Participants were recruited from a diabetes unit in a secondary care health center in a multi-ethnic East London borough of Newham. Eligible participants were insulin-requiring adults with a diagnosis of type 1 or type 2 diabetes whose most recent HbA1c was above 7.5%. Participants had to be sufficiently literate and fluent in English to complete the questionnaires and have telephone conversations with an HCP. Excluded from the study were people with prior experience using MTH , people who had not attended the clinic or had an HbA1c test done in the last 12 months, were pregnant, regularly travelled outside the UK for 3 weeks or more, required home visits by a district nurse for blood glucose (BG) monitoring and/or insulin administration, or had a diagnosis of kidney failure or sickle cell disease.

### Consent

Participants with an appointment in the following 2 weeks were screened for eligibility and sent an information sheet, an invitation to take part, and an interest form. Participants who did not refuse the invitation were approached on the day of their appointment when the nature and implications of the research were explained. Potential participants were given time to consider participating in the study and those that were willing to take part were invited to sign a consent form for the RCT. Upon completion of the 9-month RCT, separate recruitment materials were sent to the intervention group participants to inform them about, invite them to, and obtain consent for, the qualitative interviews.

### Baseline Assessment

Each participant was given the opportunity to complete the questionnaires with a researcher at the clinic or at home (with assistance, if required) using a prestamped envelope for return by postal mail.

### Randomization

Randomization occurred after the return of the baseline questionnaire. It was carried out in blocks of 20 participants using an online sequence generator. All participants were sent a letter informing them of the group they were allocated to, and intervention participants were telephoned to confirm the next steps. General practitioners were notified of their patients’ involvement in the study and allocation group.

### The Mobile Telehealth Intervention

#### Overview of the Mobile Telehealth Equipment and App

**Figure 1:**
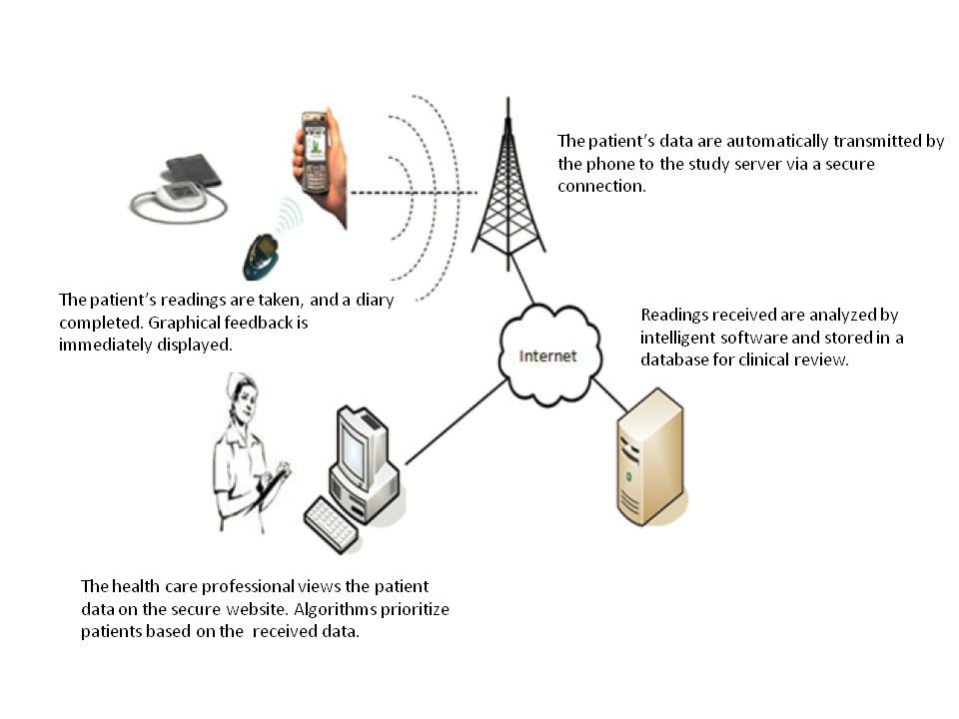
Architecture of the MTH system evaluated.

The MTH system assessed in this study was developed by a team of engineers in Oxford, England. Its design was based on earlier data transmission and diary apps, HCP advice, and user feedback, and designed according to criteria of ease of use, personalization, prompt feedback, integration to the user’s lifestyle, and quality of care [32]. The MTH equipment consisted of a mobile phone (Sony Ericsson k810i) with MTH app software installed, charger, BG meter, BP monitoring device, and Bluetooth cradle. Figure 1 represents the architecture of the MTH system. The MTH app allows for the recording of several health parameters: BG and BP readings, time since last meal, level of physical activity performed so far that day, insulin dose, and weight. BG and BP were transmitted via Bluetooth and the remaining data were manually entered using the mobile phone keyboard. The MTH app could store up to 500 clinical readings, and both single and bulk data transfers from the mobile phone to the Web server were possible. Following data recording or transfer, immediate color-coded graphical feedback was displayed on the mobile phone screen. This included representations of (1) the last BG reading recorded in comparison to the average BG reading for the last month, (2) a histogram of the frequency of BG readings within different glycaemic ranges in the last month (see ranges for glycaemic states below), and (3) scatter plots of the BG and BP readings recorded in the last 5 days. More sophisticated graphical representations of the data transferred were available via a password-protected Web account interface. Color codes represented different glycemic states: blue for hypoglycemia (0-4 mmol/L), green for normoglycemia (4-10 mmol/L), amber for borderline hyperglycemia (10-12 mmol/L), and red for hyperglycemia (above 12 mmol/L).

#### Intervention Group Protocol

In addition to receiving standard care, intervention participants were provided with the MTH equipment and a BG strip prescription letter for the BG meter. Equipment was delivered to their home by an engineer who trained them to use it. Training sessions ended when a participant was able to collect, enter, and transfer data correctly alone. On this occasion, participants were encouraged to continue to self-monitor at the frequency recommended by their HCP, and to transfer all data at every self-monitoring occasion. Weekly BP self-monitoring was recommended for participants for whom BP monitoring had not been prescribed prior to the study. Participants in the habit of relying on their BG meter for a list of readings to show their HCP were asked to attend their routine clinical appointments with the MTH mobile phone. The mobile phone could display a list of date- and time-stamped clinical readings. Alternatively, HCPs at the clinic where recruitment took place had authorized access to participants’ MTH data via a Web interface.

The intervention protocol included the MTH nurse completing the following actions:

1. Making *introductory phone calls* within 2 weeks of participants receiving MTH training to introduce herself, confirm contact details, and collect basic information on diabetes management.

2. Making *6 weekly educational calls* to deliver diabetes education. In the absence of patient-designated questions, the following topics could be covered: recognizing and managing hypoglycemia, aspects of lifestyle management in relation to alcohol, weight, smoking, food choices, physical activity, illness and diabetes, and insulin, as well as methods to optimize future diabetes routine appointments.

3. *Responding to participants’ BG and BP readings*. BG readings varied according to participants’ diagnoses (type 1 or 2) and medication (insulin and oral, or insulin only). Participants with one isolated hypoglycemic event were red-flagged for closer monitoring for 72 hours. The MTH nurse contacted participants with a borderline hyperglycemic reading and with recurring hyperglycemic and hypoglycemic events within 72 hours to assess the reasons for these low/high BG readings. Recurring hyperglycemia due to illness or medication changes was red-flagged for 24 hours for closer observation. Those with sufficient experience with insulin adjustments were encouraged to titrate their insulin dosage. Those who required a medication review were asked to schedule an appointment at the clinic. In more urgent situations (eg, possible ketoacidosis), the MTH nurse was required to advise the patient to visit an accident and emergency department. Education and reminders as to the importance of medication/lifestyle factors in the management of diabetes were to be provided as appropriate.

The MTH nurse was required to contact patients with four consecutive BP readings over 140/80 in a 14-day period to discuss medication and provide advice on lifestyle changes to improve BP. If a further four readings above 140/80 in a 14-day period occurred following a discussion with the participant on medication and lifestyle changes, the MTH nurse was required to refer the participant to a general practitioner.

The technical support team provided telephone support, organized a home visit where appropriate, and contacted participants who had not transmitted data for more than 7 days. A maximum of three successful telephone calls were made to encourage data transmission.

### Control Group

Participants in this group received standard care that consisted of a 30-minute appointment with a diabetes specialist nurse every 3 to 4 months, and 1 annual or 2 semiannual appointments with a diabetes consultant. During working hours there was at least one diabetes specialist nurse available at the clinic to receive phone calls from diabetes patients.

### Follow-Up Assessments

Participants were sent the 3- and 9-month follow-up questionnaires by postal mail with a prestamped envelope for return. Each participant was given the opportunity to complete their questionnaires with a researcher.

### Health Care Professional and Engineer Training

All diabetes specialist nurses and consultants were provided opportunities to learn how to use the MTH equipment and access the MTH data via the Web interface. The MTH nurse received training to remotely access and navigate participants’ electronic medical records. The engineer was taken through the steps to follow with participants and the content to cover in each MTH training session.

### Evaluation Measures

#### Overview

As recommended in the MRC guidance for complex interventions [23], evaluation was designed to capture information on both processes and outcomes. The quantitative study data included clinical measures, self-reported questionnaires administered to participants, logs/electronic notes from the MTH app, and records kept by the technical support team, MTH nurse, and engineer of contact with participants. For self-reported data, standardized questionnaires with good psychometric properties were used wherever possible—see Table 1 for a summary of the data collected at the three time points. Less commonly used and self-developed questionnaires are described in greater detail below. Data from the qualitative component included transcripts from audiotaped, semistructured interviews conducted with intervention participants who agreed to take part in this part of the evaluation.

#### Demographics

Data on age, gender, education, ethnicity, country of birth, and whether English was spoken at home was collected.

#### Clinical Measures

Data on diabetes type, duration, medication, complications, daily insulin dose, number of daily insulin injections, and Body Mass Index (BMI) were taken from medical records. Comorbidities were collected using self-reporting by participants.

#### Familiarity With Mobile Phones

Familiarity with mobile phones was evaluated at the time of recruitment by asking all participants who enrolled in the study whether they owned a mobile phone (yes/no).

### Primary Outcome Measure

The primary outcome measure, HbA1c, was collected from medical records.

### Secondary Outcome Measures

BP readings were recorded from medical records. Diabetes self-management behaviors were measured using the Summary of Diabetes Self-Care Activities (SDSCA) questionnaire [33], which focuses on dietary behaviors, exercise, foot care, BG testing, and cigarette smoking. An additional item asking participants to specify their weekly frequency of self-monitoring of BG levels was added to this assessment of behaviors. Generic HRQoL was assessed using the Short Form-12 (SF12v2) with a recall time of 4 weeks [34,35] and the Diabetes Health Profile (DHP-18) was used as a disease-specific measure of HRQoL [36]. Depression and anxiety were measured using the Center for Epidemiologic Studies Short Depression Scale (CESD-10) [37] and the State-Trait Anxiety Inventory (STAI-6) [38], respectively. Data on the number of appointments attended during the study at the diabetes clinic with diabetes specialist nurses and consultants were collected from medical records.

### Process Variables

Self-efficacy for managing health was measured using the Health Education Impact Questionnaire (HeiQ, v3.0) [39], which is used to investigate the impact of health interventions on patient empowerment. It consists of eight subscales that can be applied independently, two of which were used in the current study. The *self-monitoring and insight* subscale assesses an individual’s beliefs in his/her ability to monitor his/her health and the physical and/or emotional responses that lead to appropriate self-management. The *skills and technique acquisition* subscale captures the beliefs an individual has in his/her knowledge-based skills and techniques to self-manage his/her health.

Disease-specific self-efficacy was assessed using the Insulin Management Diabetes Self-Efficacy Scale (IMDSES) [40], which consists of five subscales on *general management*, *insulin management*, *dietary management*, *exercise management*, and *foot-care management*. In line with clinical practice at the diabetes unit where the study took place, references to urine testing were removed from the questionnaire (eg, the question “I cannot test my blood or urine when I am away from home” became “I cannot test my blood when I am away from home”) and an item on *food exchange* (ie, “I can correctly exchange one food for another in the same food group”) was removed, as this concept was not used in clinical practice or in diabetes education classes. Diabetes-specific illness beliefs were measured using the Personal Models of Diabetes (PMD) scale [41] consisting of two subscales focusing on beliefs related to the seriousness of diabetes and treatment effectiveness.

### Mobile Telehealth-Related Variables

#### Mobile Telehealth Self-Efficacy and Acceptability

In the absence of a currently available measure, a questionnaire was developed to capture individuals’ beliefs about their ability to operate the MTH equipment. To facilitate item generation, a researcher used the MTH system for 1 week to identify the different steps and skills required to transmit data and review feedback. Ten items were generated in relation to data entry, data transfer, menu navigation, display of graphical feedback, and use of graphical feedback to identify BG patterns and make adjustments to self-management behaviors. Each item of the questionnaire begins with “I am confident that I am able to...” The same 5-point Likert scale of another diabetes self-efficacy measure [42] was used (1=No, definitely not, 2=Probably no, 3=Maybe yes, maybe no, 4=Yes, probably, 5=Yes, definitely). Principal component analysis revealed one factor—higher scores indicated greater self-efficacy to use the MTH equipment.

At the time of this study, there was no published and psychometrically valid questionnaire to measure acceptability of MTH, therefore, a questionnaire was developed to measure acceptability. Item generation was guided by previous empirical and theoretical work on user acceptance underlining the importance of perceived usefulness, perceived ease of use, and integration into life in determining acceptability and technology usage. A principal component analysis was performed to determine subscales, and three subscales—consisting of seven items, 13 items, and seven items, respectively—were identified. Participants were asked to rate their level of agreement with each item using a 4-point Likert scale (0=Strongly disagree, 1=Somewhat disagree, 2=Somewhat agree, 3=Strongly agree). In addition to this acceptability questionnaire, individual items were included in the intervention group’s follow-up questionnaires to assess perceived adequacy (one item asking participants whether they would have liked more training) and quality of the MTH training (one item), quality of the technical support received over the phone (one item), frequency of usage of the Web interface (one item), and perceived usefulness of the Web interface (one item).

#### Participants’ Perceptions and Experiences of Use

Qualitative semistructured interviews were used to explore participants’ perceptions of, and experiences using, MTH. An interview guide was developed based on previous research and areas of interest. It addressed several topics including initial thoughts and expectations about MTH, use of the technology, perceived impact on diabetes management, the relationship with the MTH nurse, technical problems, and suggestions for improvement. To limit the influence of social desirability on responses, the relationship between the research team, the MTH provider, and HCPs was clarified before the interview to underline the independent nature of the evaluation. Participants were also reminded that there were no correct answers to the questions asked and that both negative and positive feedback was valuable to help improve the MTH service. There are no hard and fast rules about sample sizes in qualitative enquiries—the required number of interviews remains a matter of judgment and experience in assessing the quality of the data collected against the purpose of the enquiry [43]. Recruiting MTH participants for interviews continued until data saturation occurred and no new themes emerged from the data on five successive interviews. Previous qualitative TH studies included fewer than 20 patient interviews [44,45], therefore, we expected to conduct between 10 and 25 interviews.

#### Usage and Intervention Delivery

Data transmitted by intervention participants were collected as they provide some indication of intervention receipt and adherence. Data on contacts made between the MTH nurse, technical support, the engineer, and intervention participants were also collected. This included the number of contacts, the medium used (ie, telephone, text message, in person), and topic(s) discussed. To assess whether the intervention was delivered as planned (ie, intervention fidelity), these data will be compared to the fixed components of the intervention protocol (ie, one training session for each intervention participant, one introductory MTH call, six weekly educational calls, and provision of technical support to solve technical problems).

**Table 1:** Assessment protocol and data collected at the three time points.

Assessments and measurement tools used (where applicable)^a^		Data collection time point			
		Baseline	3 months		9 months
Demographics		✓			
**Clinical**					
	Type and duration of diabetes, complications, comorbidities, medication type	✓			
	HbA1c	✓	✓		✓
	BP, daily insulin dose	✓			✓
**Psychological**					
	Self-efficacy (IMDSES, HeiQ)	✓	✓		✓
	Illness beliefs (PMD)	✓	✓		✓
**Health outcomes**					
	Self-management behaviors (SDSCA, weekly frequency blood testing)	✓	✓		✓
	Quality of life (SF36, DHP-18)	✓	✓		✓
	Psychological well-being (STAI-6, CESD-10)	✓	✓		✓
Number of appointments with diabetes nurses and consultants		Over 9 months			
**MTH-related variables** ^a^					
	Acceptability of MTH (self-developed)		✓		✓
	Self-efficacy to use MTH (self-developed)		✓		✓
	Individual items on adequacy and quality of training, quality of technical support		✓		
	Individual items on Web account usage and their perceived usefulness		✓		✓
	MTH usage	Over 9 months			
**Intervention fidelity^b^**		Over 9 months			
**Patient perceptions**					
	Interview on MTH experience				✓

^a^HbA1c: hemoglobin glycated; BP: blood pressure; IMDSES: Insulin Management Diabetes Self-Efficacy Scale; HeiQ: Health Education Impact Questionnaire; PMD: Personal Models of Diabetes; SDSCA: Summary of Diabetes Self-Care Activities; SF36: Short Form Health Survey; DHP-18: Diabetes Health Profile; STAI-6: State-Trait Anxiety Inventory-6; CESD-10: Center for Epidemiologic Studies Short Depression Scale-10; MTH: Mobile Telehealth.

^b^Assessed in the intervention group only.

### Data Analysis

The section below outlines the plans for data analysis which is currently ongoing.

#### Quantitative Data Analysis

##### Effects of the Mobile Telehealth Intervention

For the primary analyses on the effectiveness of the intervention on HbA1c, intention-to-treat analyses will be conducted using hierarchical linear models as they account for the correlations between repeated measurements. A significant *Group* x *Time* interaction will be interpreted as evidence for differential treatment effectiveness. Any demographic or clinical differences at baseline will be adjusted for. These primary analyses will be supplemented with secondary sensitivity analyses including only those participants who actively transmitted MTH data during the intervention period. Separate hierarchical linear models will be used to evaluate the effects of the intervention on secondary outcomes.

##### Mechanisms of Action of the Intervention

To evaluate whether diabetes self-efficacy and illness beliefs act as mediators of change in the outcomes, bootstrapping for mediation analyses using Preacher and Hayes macros [46] will be conducted using residualized change scores for relevant process and outcome variables.

##### Predictors of Usage

Hierarchical linear regressions will be conducted to examine the incremental contribution of baseline predictors and MTH-related variables in the prediction of MTH usage. Telehealth-related variables considered as potential predictors are based on the extended TAM model used in this study and include perceived usefulness, ease of use, integration into life, adequacy of training, and self-efficacy to use MTH.

#### Qualitative Data Analysis

A step-by-step guide for thematic analysis [47] will be followed to analyze the interview data. Transcripts will first be read several times to become familiar with the data. Initial coding of the interviews will follow. The data will then be organized into themes and subthemes. The approach used will be inspired by the constant comparative method used in grounded theory and its combined elements of induction and deduction. This hybrid approach allows for themes identified in previous research to be considered during analysis, but also allows for unexpected findings to emerge from the transcripts. To improve the validity and reliability of the analysis, several researchers with previous experience in MTH and/or qualitative data will independently code some of the transcripts in order for themes to be compared and/or will participate in discussions on the extracted themes and supporting quotations.

## Results

### Overview

At this stage, data collection for both the quantitative and qualitative components of this study has ended and data analysis is ongoing. A total of 86 participants were enrolled into the study. Out of 86 participants, 45 (52%) were randomized to the intervention group and 36 (42%) to the control group. In this paper, the data presented on MTH training sessions are relative to the 44 intervention participants who received training. Data presented on other MTH-related variables including MTH usage, technical problems, and technical support experience are for the 40 intervention participants who completed the 9-month intervention.

### Mobile Telehealth Training Sessions

Of the 45 participants allocated to the intervention group, 44 (98%) received training in the use of the MTH equipment (1 participant dropped out prior to finding out his allocation group). The majority of trained intervention participants (37/44, 84%) were able to transmit MTH data after the initial training session. A small group of those trained (7/44, 16%) required a second training session after experiencing ongoing difficulties in using the equipment correctly. Compared to the 37 intervention participants who only required one MTH training session—mean age 56.4 years (SD 13.9), 22% (8/37) with no formal education—these 7 participants were older and less educated—mean age 67.5 years (SD 8.6), 43% (3/7) with no formal education. These differences were not tested statistically given the small number of participants involved. At the 3-month follow-up, 17 (39%) of the 44 trained MTH participants reported they would have liked to receive more training. When asked at 3 months about the quality of the training provided, 29 (66%) of the 44 participants rated the quality of the training to be *good* or *very good*, 10 (23%) rated the quality as *adequate*, and 2 (5%) as *insufficient*.

### Mobile Telehealth Usage

Table 2 describes the number of times participants transferred data during the trial, as well as the number of clinical readings (ie, BG and BP readings) transmitted. The monthly number of data transfers ranged from 0 to 126 and the median number of transfers over 9 months was 63 (interquartile range [IQR] 242, mean 173.9, SD 232.8). Of the 40 participants, 10 (25%) of them were particularly active and transferred data between 202 and 778 times over 9 months. The monthly number of BG readings transmitted ranged between 0 and 186, and the median number of transfers over 9 months was 147 (IQR 337, mean 251.7, SD 278.0). In comparison to BG levels, BP was monitored less frequently. The monthly number of BP readings transmitted ranged between 0 and 66, and the median number of BP readings transmitted over 9 months was 19 (IQR 31, mean 33.6, SD 53.3).

Table 3 displays the timing at which BG readings were self-monitored.

Over the 9 months of the study, the median number of times physical activity and insulin dose data were transferred was 11.5 (IQR 60) and 15.0 (IQR 83), respectively. This corresponded to 40.8% (71/174) and 55.7% (97/174) of data transfer occasions for physical activity and for insulin dose, respectively. Weight information was rarely updated, with the average number of updates over 9 months being 4.08 (SD 9.47) times. A large proportion of participants (17/40, 43%) never updated their weight information.

**Figure 2:**
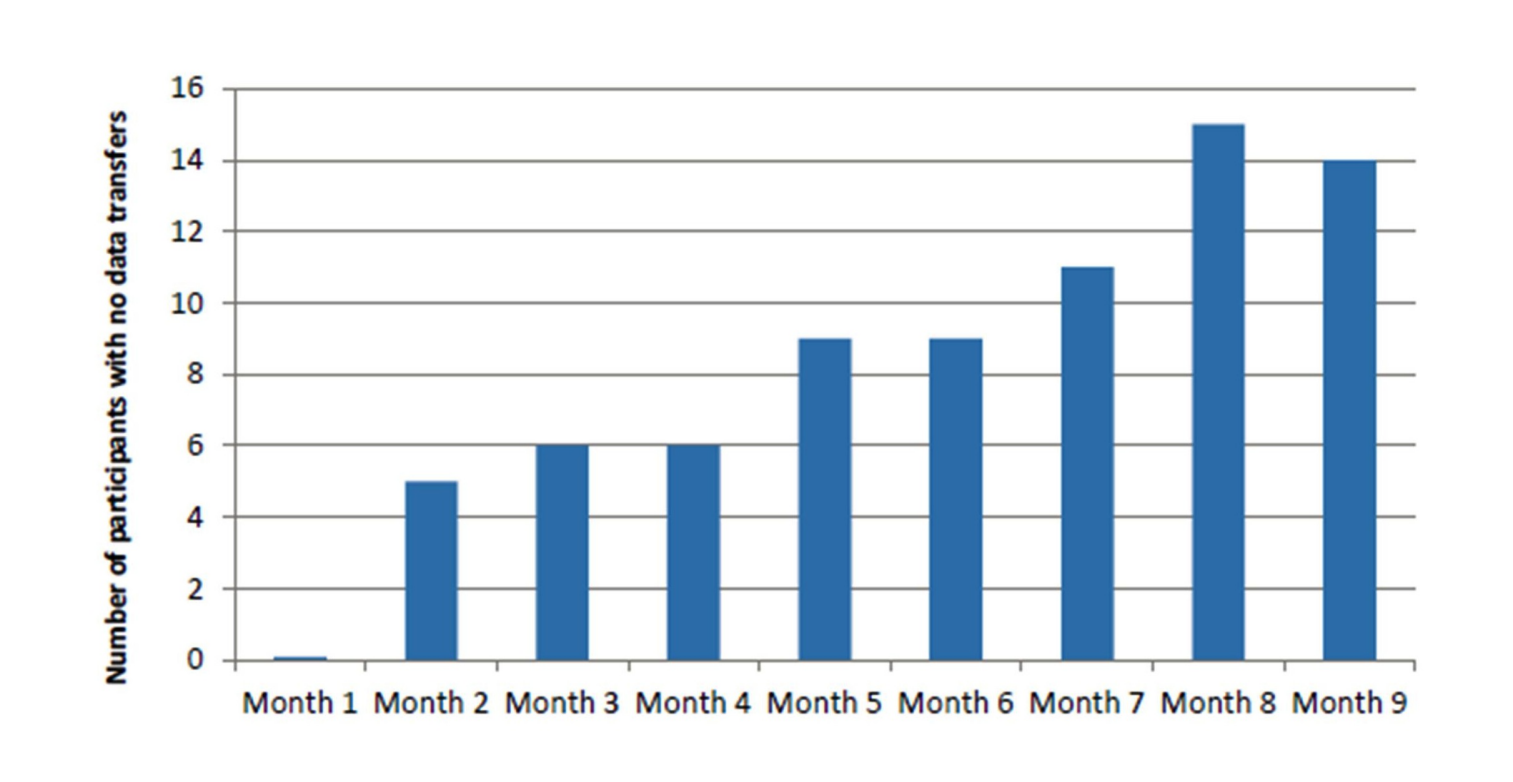
Number of participants per month who did not transmit mobile telehealth data during the study.

Table 2 indicates that for all measures of MTH, usage decreased over time. The number of participants who did not transmit any data increased over the duration of the trial as seen in Figure 2. In the first month, all participants transmitted some MTH data. By month 9, there were 14 out of 40 (35%) intervention participants who did not transmit any MTH data.

Table 4 describes the different durations during which participants ceased to transfer MTH data. The majority of participants (22/40, 55%) transferred MTH data at least once every month. For the remaining participants, the number of months during which no data was transferred ranged from 1 to 8, with the majority of these participants transmitting data for at least 5 of the 9 months.

The Web interface was available to all participants, however only 5 (13%) and 6 (15%) of the 40 participants reported using the MTH Web interface at the 3- and 9-month follow-ups, respectively. The 5 participants that reported using the Web account at 3 months also reported using it at 9 months. Of the participants that used the Web interface at 3 months, 80% (4/5) reported weekly usage at this time point. Of the participants that used the Web interface at 9 months, 83% (5/6) reported monthly usage at this time point. All participants who used the Web account during the study reported that it was *quite a bit useful* or *very useful* for visualizing graphical feedback.

**Table 2:** Number of MTH data transfers and clinical readings transmitted during the 9-month study.

Month in trial	Number of data transfers, mean (SD)	Number of BG readings, mean (SD)	Number of BP readings, mean (SD)
Month 1	25.9 (34.2)	31.2 (37.5)	6.6 (10.5)
Month 2	23.4 (30.9)	31.4 (35.9)	5.1 (9.1)
Month 3	22.6 (31.5)	31.2 (37.0)	5.0 (9.6)
Month 4	20.8 (28.0)	27.9 (30.3)	3.3 (5.8)
Month 5	18.0 (20.5)	26.2 (27.6)	3.6 (6.0)
Month 6	16.3 (25.8)	26.7 (30.0)	3.4 (4.5)
Month 7	15.9 (26.0)	24.6 (31.9)	3.1 (5.7)
Month 8	16.1 (27.3)	28.6 (34.8)	2.2 (4.2)
Month 9	14.9 (25.2)	24.6 (33.3)	2.1 (3.9)
Over 9 months	173.9 (232.8)	251.7 (278.0)	33.6 (53.3)

**Table 3:** Timing of self-monitoring of BG levels.

Timing in relation to meal	Number of BG readings (n=6959), n (%)
>8 hours after a meal^a^	1302 (18.71)
2-4 hours after a meal	1852 (26.61)
0-1 hour after a meal	1512 (21.73)
1-2 hours after a meal	969 (13.92)
4-8 hours after a meal	1324 (19.03)

^a^BG readings taken >8 hours after a meal are likely to be fasting BGs.

**Table 4:** Length of time during which participants transferred no data during the study (n=40).

Number of months during which no data was transferred	Proportion of MTH participants, n (%)
0	22 (55)
1	2 (5)
2	2 (5)
3	6 (15)
4	1 (3)
5	2 (5)
6	1 (3)
7	1 (3)
8	3 (8)

### Mobile Telehealth Technical Problems

In total, 26 of the 40 participants (65%) experienced technical problems during the study. The problems signaled were related to data transmission between devices not working properly (13 occurrences), faulty equipment or problems with equipment settings (10 occurrences), batteries on the mobile phone, BG meter, or Bluetooth cradle running out (12 occurrences), problems with the MTH app (3 occurrences), Web account log-in problems (2 occurrences), and step-by-step assistance to transmit MTH data required (8 occurrences). With the exception of complaints about the battery life in the mobile phones (3 occurrences), all technical problems were successfully resolved over the telephone or during a home visit scheduled within 1 week by the engineer on 6 occasions. Replacements for faulty equipment were sent through postal mail. The MTH Web server was down once during the study and repaired within 24 hours by the technical support team. The majority (19/22, 73%) of participants who reported having received technical support over the phone at 3 months indicated it was of *good* or *very good* quality.

## Discussion

This study proposes a comprehensive assessment of an MTH intervention for people with insulin-requiring diabetes. The measurement of both clinical and patient-reported outcomes, the use of a qualitative enquiry alongside an RCT, and the focus on intervention delivery and usage still remain relatively uncommon in complex interventions [20,24,48] and are included in this study’s design and scope.

The preliminary data presented in this paper shows that the initially targeted sample size of 248 was not reached. Several reasons may help explain the low participation rate experienced in this study, including poor attendance to clinic visits, changes to the clinic patient discharge policy, and recruitment to other TH trials at the diabetes unit where our recruitment took place—the recruitment challenges experienced in this study will be discussed in another paper. Such recruitment difficulties are not uncommon in TH trials [49,50].

The early data presented on MTH usage in this paper clearly indicates a decrease in MTH usage over time. Gradual declines in usage have been observed in other TH studies [51-53]. A positive interpretation of these declines over time proposed by Larsen et al [52] is that participants may become less dependent on MTH because of perceived improvements in the management of their condition. Another possible explanation for the decrease in usage over time is that the novelty of the new technology wears off. Mobile phone network coverage is unlikely to have influenced MTH usage in this study as coverage is generally good in the UK and was not one of the problems the MTH participants reported experiencing. Other technical problems occurred, but our data showed they were dealt with successfully and promptly by the technical support team. Making sure that satisfactory technical support is provided in MTH studies is key as technical problems can result in increased dropouts and negative attitudes toward MTH [54].

The Web component of the MTH system remained unused by a large majority of the intervention participants (34/40, 85%) who completed this study. Participants were informed about the possibility of using the Web accounts at the beginning of the study. However, the MTH training sessions did not include instructions relating to the MTH Web accounts, therefore, this is likely to have contributed to their low use. Low Web account usage in this study may also have been related to the lack of Internet access in 63% of households in Newham [55], low perceived need of access or usefulness of this data, disinterest, or lack of awareness of the existence of a Web component.

Our study data showed that 39% (17/44) of MTH participants reported they would have liked to receive more training. Other studies have highlighted that some participants using TH may require a period of adjustment and familiarization with technology, and that TH may be associated with technology-related anxiety [56,57]. Together with our findings, these studies suggest that some MTH users may benefit from attending more than one training session. Of the 44 MTH participants trained in our study, 7 (16%) were unable to use the MTH equipment correctly and required a second training session. Their characteristics suggested that factors such as age and educational attainment may be related to training requirements, however these relationships were not investigated statistically, given the small number of participants concerned. A small amount of research has examined factors related to TH usage compliance [51,58], but there has been little emphasis on predictors of MTH training needs. Larger studies should aim to identify the individual characteristics associated with greater MTH training needs, which could further help improve the tailoring of MTH interventions.

This paper provides details of MTH usage data and other information collected in this study on the quality and adequacy of the MTH training sessions, and on the technical problems experienced by MTH participants. Few MTH studies provide sufficient information on technology usage, despite this being an important measure of participants' receipt and adherence to the intervention. The data presented in this paper are related to intervention fidelity and are, therefore, crucial in considering the internal validity of the study.

## Abbreviations

BG: blood glucose
BMI: Body Mass Index
BP: blood pressure
CESD-10: Center for Epidemiologic Studies Short Depression Scale
DHP-18: Diabetes Health Profile
HbA1c: glycated hemoglobin
HCP: health care professional
HeiQ: Health Education Impact Questionnaire
HRQoL: health-related quality of life
IMDSES: Insulin Management Diabetes Self-Efficacy Scale
IQR: interquartile range
MRC: Medical Research Council
MTH: mobile telehealth
NHS: National Health Service
PMD: Personal Models of Diabetes
RCT: randomized controlled trial
SDSCA: Summary of Diabetes Self-Care Activities
SF12v2: Short Form-12
STAI-6: State-Trait Anxiety Inventory
TAM: Technology Acceptance Model
TH: telehealth
